# Open arch—my way! Branch first continuous perfusion arch repair (BF-CPAR)

**DOI:** 10.1007/s12055-023-01535-2

**Published:** 2023-07-17

**Authors:** George Matalanis, Varun J. Sharma

**Affiliations:** 1 Brian F. Buxton Department of Cardiac and Thoracic Aortic Surgery, Austin Health, Melbourne, Australia; 2 Department of Surgery (Austin Health), Melbourne Medical School, The University of Melbourne, Melbourne, Australia

**Keywords:** Aortic arch surgery, Branch first continuous perfusion

## Abstract

We herein describe our technique of “branch first continuous perfusion arch repair (BF-CPAR)” which does away with both cerebral circulatory arrest and the need for deep hypothermia. We use this technique for all aortic surgeries including for type A acute aortic dissections.

**Supplementary Information:**

The online version contains supplementary material available at 10.1007/s12055-023-01535-2.

We herein describe our “branch first continuous perfusion arch repair (BF-CPAR)” for open arch repair which we have been performing for two decades. I began performing a technique for total arch replacement which did away with both cerebral circulatory arrest and the need for deep hypothermia [1–10]. This procedure is the preferred strategy for all patients undergoing aortic surgery at our institution, including for acute type A aortic dissections.

Arch branch reconstruction takes the highest precedence in this procedure, to secure cerebral blood flow early in peace [1–4, 6, 7]. Secondly, neither the heart nor the rest of the circulation are penalized unnecessarily with ischemia during this phase. Each arch branch in turn is isolated between clamps, anastomosed to a branch of the trifurcation graft, and after careful de-airing, then reperfused. The period of isolation is short, on average 10 min for the innominate artery (IA) and left common carotid artery (LCCA), and 15 min for the left subclavian artery (LSCA). Key points include:

1. Specific modification of the Spielvogel trifurcation graft with a carefully chosen position of side perfusion port to work logically with the sequence.
2. Dedicated “head pump” to allow exact control over cerebral flow.
3. Reconstruction sequence of the following: IA, LCCA, LSCA. As each artery is debranched, greater access is gained to the next deeper artery.

A possible concern would be substituting focal for global ischemia, in patients where the circle of Willis is inadequate. We emphasize that the circle of Willis (CoW) is the main collateral source in carotid clamping at its bifurcation in the neck as would be done for a carotid endarterectomy. However, clamping an arch branch near its origin is entirely different. There are numerous collaterals across the midline of the head and neck, around the shoulder girdle, and between the upper and lower body along the trunk. These provide voluminous flow to the clamped territory way above that of the CoW.

Apart from no global cerebral circulatory arrest, additional advantages of BF-CPAR are:

- Shorter myocardial ischemia time: arch branches already done before antegrade cerebral perfusion.
- Shorter distal organ ischemia time: nil if there is a distal clamp, one anastomosis time if distal circulatory arrest.
- Modular arrangement: arch branch reconstruction out of operative field until the last stage allows better quality tissue by avoiding “island” technique, all anastomoses in easy reach even after off bypass.
- Moderate hypothermia: it reduces bypass time and consequent coagulopathy.

After sternotomy, and prior to institution of bypass, innominate vein and arch branch mobilization is performed as this substantially reduces bypass time. The innominate vein is preserved, to avoid the potential for left cerebral hemispheric or left arm venous edema. The risk of inadvertent tears to the vein on subsequent re-sternotomy is mitigated by reconstituting the thymic fat pad over it before sternal closure. It is important to mobilize the arteries with minimal disturbance to the arch itself or its branches to avoid the risk of athero-embolization. It is frequently possible to free the whole IA and LCCA, and sometimes even the LSCA before bypass.

Central cannulation is used whenever feasible. Alternatively, femoral cannulation with pre-deployment of vascular closure devices (Pro-glide) can be used unless the computed tomography (CT)-aortogram demonstrates aortoiliac atheroma at risk of retrograde embolism, in which we case we use axillary cannulation. We start with IA reconstruction. The heart remains perfused and beating, reducing cardiac ischemia time. It is important to measure the trifurcation branch lengths under good stretch to avoid subsequent kinking when the graft is fully pressurized. A thorough de-airing sequence is followed before re-instituting antegrade perfusion to the reconstructed branch. Finally, the innominate stump is ligated with the innominate branch out of the way, so access to the LCCA for clamping and anastomosis is much easier. It is important to perform this anastomosis on the same side of the innominate as the first anastomosis to avoid sandwiching it. Greater precision is required with this anastomosis in small and thin-walled carotids especially in females to avoid stenosis and we prefer 6/0 instead of 5/0 prolene in this case. With the 2 previous branches out the way, better access to the usually deep LSCA is gained.

There are several tricks to further improve access. If the arch pathology and future distal aortic interventions allow it, then the LSCA can be left alone. Other maneuvers include short extension of the skin incision into the neck along the left sternomastoid border, an assistant providing gentle upward leverage on the distal LSCA clamp, lowering distal aortic pressure, or even deferring LSCA until distal aortic decompression. Note how the trifurcation graft sits beautifully out of the central operative field while the frozen elephant trunk (FET) is completed with a brief period of distal circulatory arrest after which distal perfusion is restarted and proximal anastomosis is performed. Finally, the trifurcation graft is anastomosed to the first branch of main graft still without interruption to cerebral perfusion.

### Supplementary Information

Below is the link to the electronic supplementary material.

Supplementary file1 (mov 218 MB)
